# Surgical management of extrahepatic portal vein obstruction in children: advantages of MesoRex shunt compared with distal splenorenal shunt

**DOI:** 10.1007/s00383-023-05411-3

**Published:** 2023-02-16

**Authors:** Omar Khamag, Alp Numanoglu, Heinz Rode, Alastair Millar, Sharon Cox

**Affiliations:** Division of Paediatric Surgery, Red Cross War Memorial Children’s Hospital and University of Cape Town, Klipfontein Road, Rondebosch, Cape Town, 7700 South Africa

**Keywords:** Portal hypertension, Extrahepatic portal vein occlusion, Variceal bleeding, Portosystemic shunts, Hepatobiliary surgery, MesoRex shunt

## Abstract

**Purpose:**

To review surgical management of extrahepatic portal vein obstruction (EHPVO) at Red Cross War Memorial Children’s Hospital and compare MesoRex shunt (MRS) with distal splenorenal shunt (DSRS).

**Methods:**

This is a single-centre retrospective review documenting pre- and post-operative data in 21 children. Twenty-two shunts were performed, 15 MRS and 7 DSRS, over an 18-year period. Patients were followed up for a mean of 11 years (range 2–18). Data analysis included demographics, albumin, prothrombin time (PT), partial thromboplastin time (PTT), International normalised ratio (INR), fibrinogen, total bilirubin, liver enzymes and platelets before the operation and 2 years after shunt surgery.

**Results:**

One MRS thrombosed immediately post-surgery and the child was salvaged with DSRS. Variceal bleeding was controlled in both groups. Significant improvements were seen amongst MRS cohort in serum albumin, PT, PTT, and platelets and there was a mild improvement in serum fibrinogen. The DSRS cohort showed only a significant improvement in the platelet count. Neonatal umbilic vein catheterization (UVC) was a major risk for Rex vein obliteration.

**Conclusion:**

In EHPVO, MRS is superior to DSRS and improves liver synthetic function. DSRS does control variceal bleeding but should only be considered when MRS is not technically feasible or as a salvage procedure when MRS fails.

## Introduction

Portal hypertension (PH) is a common complication of chronic liver and portal vein pathology in children. It is defined as a pathological increase in the pressure of the portal venous system [1]. There is no clear consensus on the normal portal pressure for the different age groups. Nevertheless, an agreement exists that any increase beyond 11 mmHg is considered pathological and, once established, will have significant morbidity and mortality [2].

Two leading causes for PH in children, pre and post-sinusoidal liver disease, and pre-hepatic non-cirrhotic portal vein occlusion, also referred to as extrahepatic portal vein obstruction (EHPVO) [3]. Omphalitis, neonatal sepsis, repeated abdominal infections, sepsis, abdominal surgery in childhood, neonatal umbilical vein catheterisation, and trauma are associated with EHPVO [4]; however, the underlying aetiology of EHPVO in children remains poorly comprehended, and almost half of the reported cases are idiopathic.

Management of EHPVO in the paediatric population is primarily medical, and control of variceal bleeding by sclerotherapy or banding is an essential initial management strategy. Shunts are reserved for those who need them, with non-physiological (DSRS) and physiologic (MRS) shunts representing a safe and effective method for the long-term management. Several cohort studies have demonstrated significant improvements in growth parameters following shunt surgery, and thus surgical intervention should be actively considered in selected children presenting with PH [5]. Although different shunts have been proposed for EHPVO, both the MesoRex MRS (physiologic) shunt and distal splenorenal shunt (DSRS) (non-physiologic portosystemic shunt) have shown the most promising results as effective and definitive approaches to alleviating EHPVO. These procedures can reverse PH manifestations with low rates of post-operative morbidity and mortality [6].

Most patients present initially with nearly normal liver function only to deteriorate with time [7–9]. The symptoms associated with EHPVO are twofold: those secondary to diminished portal blood flow to the liver and those resulting from portal hypertension and spontaneous portosystemic shunting as evidenced by upper GIT bleeding.

The deranged liver enzymes in EHPVO are referred to as portal biliopathy and are proposed to arise from the impact of portal venous obstruction with reduced flow to the liver which results in long-term cholestasis and mildly elevated transaminases [10, 11] and acceleration in the liver ageing [12–14]. A prospective study of 61 paediatric patients with EHPVO observed both impaired liver growth as well as physical growth stunting in (51%) across the cohort [15].

Several explanations have been proposed for these observations, including the fact that reduced portal flow deprives the liver of stimulatory hepatotropic effects of splanchnic hormones [13, 16, 17] and hepatotropic substances that regulate liver growth and function from the intestine and pancreas [13].

A well-developed collateral circulation between the cavernoma and the intrahepatic portal system results in a less pronounced hepatic dysfunction [18]. Despite this, hepatic synthetic function remains affected in EHPVO. This usually presents as variable degrees of impaired coagulation characterised by an elevated serum prothrombin time (PT) and/or partial thromboplastin time (PTT), international normalised ratio (INR), and low levels of albumin, fibrinogen, and reduced detoxification by the liver which may result in elevated serum ammonia [19].

The clinical diagnosis of extrahepatic portal hypertension (EHPH) secondary to EHPVO is principally defined by the symptomatology resulting from increased pressure in the portal venous system in the presence of an otherwise normal or preceded by mildly deranged liver function [20]. Clinically, it is manifested as oesophageal and/or gastric varices, foregut bleeding, splenomegaly, hypersplenism, growth retardation and/or neurocognitive impairment [21].

The diagnosis of EHPVO is confirmed with Doppler ultrasonography, contrast-enhanced computed tomography and magnetic resonance angiography of the portal venous system [22], in addition to the upper gastrointestinal endoscopy to look for the source of haematemesis or melena.

**Fig. 1:**
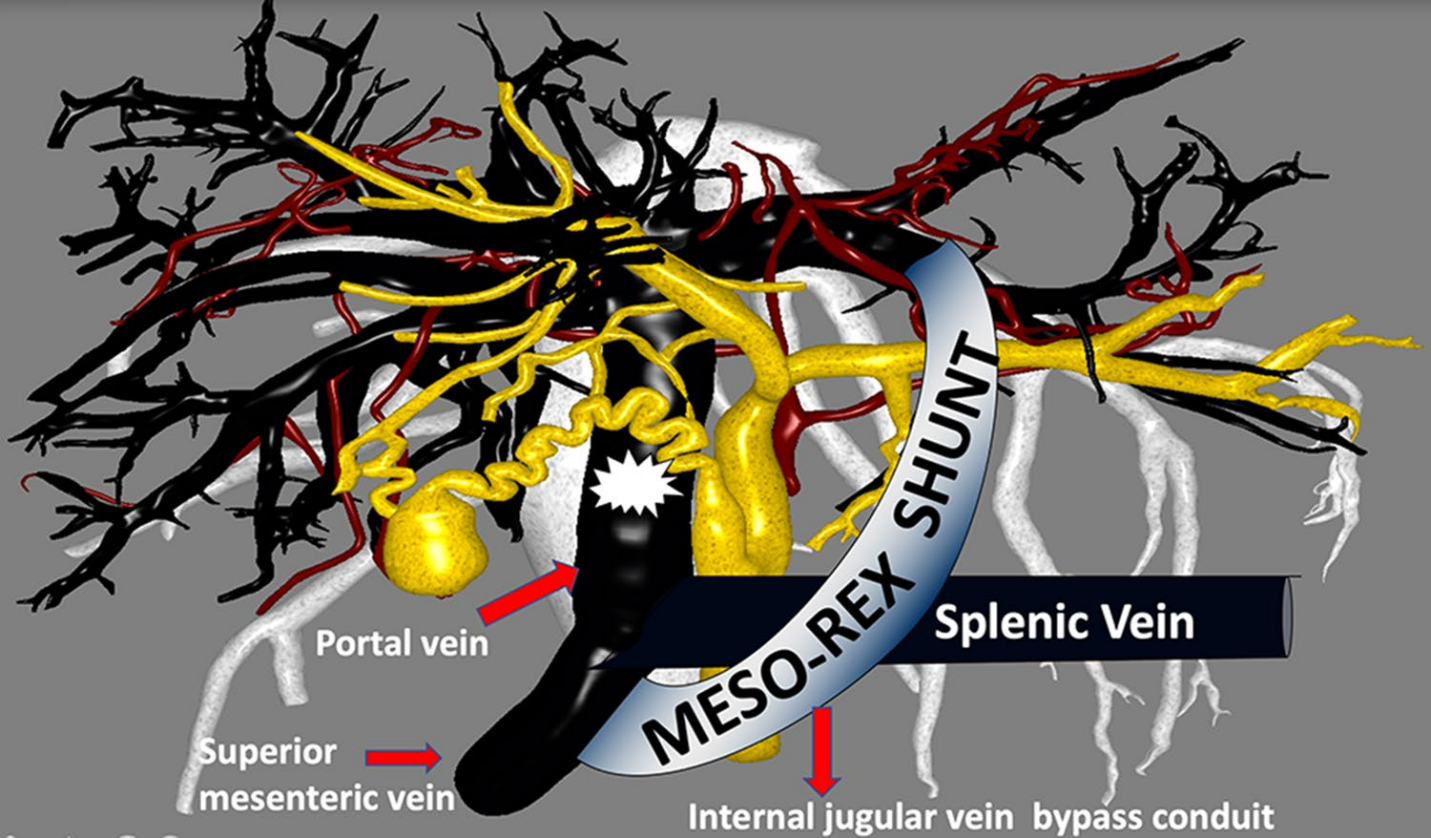
MesoRex shunt anatomy

The management of EHPH in children has evolved over several decades from variceal sclerotherapy or banding to non-selective portosystemic shunts (mesocaval and portocaval), to selective DSRS and eventually to the physiologic MRS [23]. While DSRSs partially divert blood away from the liver to the systemic circulation [24], MRSs restore the hepatopetal blood flow [25], (Fig. 1).

The portal blood flow represents half of the total hepatic blood inflow and 40% of the hepatic oxygen supply. Shunting this volume partially or completely away from the liver through the natural development of portosystemic shunts or surgically created portosystemic shunts will deprive the liver of this flow either partially or fully. Surgical portosystemic shunts may improve portal hypertension and resolve resultant upper GIT bleeding and may reduce the splenic size and the leukopenia and thrombocytopenia of hypersplenism. However, they redirect blood away from the portal circulation [26] exacerbating liver dysfunction and hepatocyte injury due to accelerated apoptosis [27].

The definitive surgical procedure to resolve EHPVO, provided the Rex vein is patent, is to perform the physiological mesenteric-left portal bypass also known as MesoRex shunt which was first described in 1996 [25]. The MRS restores hepatopetal blood flow by insertion of a venous graft between the superior mesenteric vein and the end of the intrahepatic left portal vein branch in the Rex fossa [28] to restore venous inflow to the liver. MRS results in liver growth and restores the liver mass to normal [29, 30].

This approach, the MRS, can be as effective as portosystemic shunts in the prevention of gastrointestinal (GI) bleeding and avoids their side effects, including hepatopulmonary syndrome (arteriovenous shunting), portopulmonary syndrome (pulmonary hypertension), hepatic encephalopathy, and liver nodules [31, 32] and has additional metabolic benefits including the reversal of portopulmonary syndrome encountered in established EHPVO [33, 34].

## Aims

Our study aimed to determine and compare the outcome and shunt success rate of the patients operated on in our unit and underwent either MRS or DSRS. Shunt success is defined as shunt patency at 24 months of the MRS and DSRS. Moreover, to study the possible additional benefits of the MRS over the DSRS. The effect of these two shunts on the long-term synthetic liver function and variceal bleeding and the factors that could have influenced the patency of the Rex vein was also investigated.

We hypothesised that the MRS would be superior to the DSRS in terms of patency and function with lower complications related to MRS.

## Methods

This is a single-centre, retrospective study of children who underwent either MRS or DSRS for EHPVO over an 18-year period (2001–2019).

Data on patient demographics, including age, gender, aetiology, preoperative symptomatology, Rex vein patency, age of shunt surgery, shunt patency and resolution of symptoms were collected. Additionally, platelet count, PT, PTT, INR, fibrinogen, albumin, total bilirubin, and liver enzymes prior to and two-year post-shunt surgery for both MRS and DSRS groups were compiled. Rex vein patency was assessed preoperatively with wedged hepatic vein portography, Doppler ultrasound, CT angiography, conventional angiography, or direct exploration at the time of surgery [35].

All information was obtained from patient records, Division of Paediatric Surgery surgical procedures data base, Radiology PACS system and the National Health Laboratory service patient results portal.

The standard preoperative assessment methods and the surgical technique described by De Ville De Goyet for MRS [33], and by Warren for DSRS [32], were utilised. Decisions relating to which shunt was to be performed were related to demonstration of a patent rex vein on either surgical exploration, Doppler ultrasound and direct angiography, CT angiogram or wedged hepatic vein portography, our investigation protocol to assess patency evolved over time in the stated order. All patients deemed to have a patent Rex vein on preoperative imaging or exploration underwent a MRS provided they have patent superior mesenteric vein and adequate size venous conduit (in our study Left internal jugular vein was the preferred conduit), while those found to have no patent rex vein on pre operative investigation and exploration received DSRS.

### Inclusion and exclusion criteria

All patients presented to RCWMCH with EHPVO over an 18-year period (2001–2019) were eligible for inclusion either for MRS or DSRS. Exclusion criteria included patients lost to follow-up, patients who had atypical shunts not falling into either the DSRS or MRS operation and those with insufficient or missing clinical records over 18 years.

### Criteria for MRS

1. EHPVO with patent superior mesenteric vein
2. Patent Rex vein
3. Patent right and left internal jugular vein, as one of them (preferably the left internal jugular vein) will be utilised as an interposition venous graft.

### Criteria for DSRS

1. Obliterated Rex vein.
2. Thrombosis extending to the superior mesenteric vein.
3. Patent splenic vein
4. Patent left renal vein

The existing hospital post-operative protocol was followed for patients undergoing surgery. The in-hospital post-operative regime consisted of prophylactic subcutaneous low molecular weight heparin at 0.5 mg/kg daily. On day four acetylsalicylic acid was started at a 1 mg/kg/day and continued for six to twelve months. Doppler ultrasonography to establish shunt patency was performed routinely to assess shunt patency on days zero, one, three and seven, then at six weeks and continued quarterly for a year and then annually. Shunt success was defined as a minimum flow rate of 20 cm/second for a least 24 months post-operatively and cessation of further upper GIT bleeding [36]. Esophagogastroduodenoscopy was performed only in the event of further upper gastrointestinal bleeding. In addition to a routine annual clinical assessment, full blood count and liver function tests were performed.

Statistical analysis was performed using the SPSS software package. Basic descriptive statistics were used to characterise and compare the patient cohort, including mean values, standard deviations, ranges, and percentages. Liver function and platelet values were assessed with contingency tables. This included Chi-squared statistics (${\mathrm{\rm X}}^{2}$), Fisher’s exact test and Mann–Whitney-U-Test. A two-tailed test p-value of less than 0.05 was deemed significant.

All patients presenting to RCWMCH with EHPVO over an 18-year period (2001–2019) were eligible for inclusion either for MRS or DSRS. Exclusion criteria included patients lost to follow-up, patients who had atypical shunts not falling into either the DSRS or MRS operation and those with insufficient or missing clinical records.

Ethical permission was obtained from the Human Research and Ethics Committee/University of Cape town / HREC REF 107/2019.

## Results

A total of 23 patients with EHPVO were recorded during the study period. The average age was 5 years (range 2–12), with a male to female ratio of 1.1:1 (12 M, 11F).

Fourteen patients (60%) gave a history inclusive of a risk factor for EHPVO: 5 patients (22%) had a history of umbilical vein catheterization (UVC) as an infant, one patient (5%) had a history of abdominal tuberculosis with periportal lymphadenitis in addition to UVC, two patients (9%) had omphalitis, five patients (22%) had a liver transplant, and one patient (5%) had abdominal TB. The remaining nine patients (39%) were deemed idiopathic EHPVO as no risk factors were identified.

Two patients (9%) were lost to follow-up soon after a diagnosis of EHPVO was established, and both excluded. Amongst the remaining 21 children with EHPVO, all showed increased splenic size at presentation. Only one child presented with splenomegaly, leukopenia, and thrombocytopenia (hypersplenism). All others presented with upper GIT bleeding, either due to gastric varices in 2 (10%) or secondary to oesophageal varices in the others (90%). There was an overall average of 4 episodes of variceal bleeding (range: 1–7). Further bleeding was controlled with either injection sclerotherapy in 17 (80%), band ligation in 2 (10%) or both in one child (5%). Complications reported for children who underwent sclerotherapy included 2 (10%) mild oesophageal strictures that resolved spontaneously, 4 (20%) children experienced temporary dysphagia and 8 (40%) reported retrosternal chest pain immediately post sclerotherapy.

In all patients, the splenic, superior mesenteric vein and left renal vein were patent on doppler ultrasound. Wedged hepatic vein portography (WHVP) showed a patent Rex vein in 15 patients (70%), and an occluded Rex vein in 6 (30%) patients. Intraoperatively, and during Rex vein exploration, one child who showed patent Rex vein on WHVP turned to have an obliterated vein on exploration, and one was flagged as having obliterated vein turned to have a patent Rex vein. The imaging pattern on WHVP provided an overall sensitivity of 80% and a specificity of 95% in predicting the Rex vein patency.

The fifteen children with a patent Rex vein subsequently underwent MRS surgery. The left internal jugular vein was utilised in all MRSs as the interposed conduit except one. This child had a reduced size liver transplant post Budd–Chiari syndrome, presented with EHPVO one year later. Intraoperatively, during MRS surgery, the superior mesenteric vein was dilated and tortuous. A direct side to side anastomosis between the superior mesenteric vein and Rex vein was performed. A successful MRS was demonstrated for this child on subsequent follow-up. One child was salvaged with a DSRS due to early MRS thrombosis. The onset of this was within 12 h post-surgery. The 6 children with a non-patent Rex vein (5 on preoperative imaging and one at exploration) received a DSRS, adding to that the child who was salvaged with DSRS, we ended up with 7 children in DSRS cohort.

Twenty-one patients were followed up long term, with 14 patients (66%) in the MesoRex shunt group and 7 patients (33%) in the DSRS group, including the child who had a salvage splenorenal shunt. The average follow-up in the MRS cohort was 136 months (22–218 months) and 129 months (82–188 months) in the DSRS group (*P* = 0·689). Fourteen of the 15 MesoRex procedures (93%) were deemed successful in comparison to 5 out of 7 (73%) in the DSRS group (*P* = 0·001). One of the 15 children who received a Rex shunt had reduced shunt flow (15 cm/second) at 6 months, resulting in two episodes of upper GI bleeding. This, however, on further follow-up, resolved spontaneously without further intervention needed.

Amongst the 7 children who received a DSRS shunt, 2 presented with ongoing symptoms of upper gastrointestinal bleeding. Doppler Imaging showed impaired shunt stream, which required further sclerotherapy and anticoagulation. In addition, one of these children developed marked splenomegaly, with the family refusing further surgical intervention.

Two-year post-shunt surgery, all children successfully treated with either MRS or DSRS showed reduced splenic size. The exact size reduction was not quantified in this study as measurements were not in standard planes throughout the study period.

Pre-operative bloods and blood results at the 2-year post-operative follow-up were analysed for the two cohorts. Among the MesoRex shunt group, the statistical analysis showed a significant improvement in the serum albumin, with this increasing from a mean of 32.87 g/dl preoperatively to 39 g/dl 2 years post-MesoRex surgery (*P* = 0.025). PT and PTT showed a significant change from a mean of 14.6 to 12.6 (*P* = 0.04) and from 35.6 to 31.7 (*P* = 0.018), respectively. In addition to the significant improvement in the platelet count from a mean value of 98.13 preoperatively to 182.07 2 years post-operatively (*P* = 0.01). There was also a mild improvement in the serum fibrinogen from a mean value of 2.04 g/dl–2.44 g/dl; however, this was not statistically significant (*P* = 0.15). Table 1 depicts the liver function and platelet values prior to and 2 years post-shunt surgery for both the MesoRex and DSRS.

**Table 1:** Liver function and platelet values prior to and 2 years post-shunt surgery for both MRS and DSRS

Value	Pre-operative (mean)		Post-operative (mean)		*P* value	
	MRS	DSRS	MRS	DSRS	MRS	DSRS
Albumin	32.8	31.0	39.0	32.16	0.025	0.61
PT	14.6	14.39	12.6	13.6	0.04	0.53
PTT	35.6	34.0	31.79	39.56	0.018	0.18
INR	1.9	1.27	1.2	1.2	0.24	0.42
Fibrinogen	2.04	2.14	2.44	2.12	0.15	0.87
Total bili	17.93	10.6	14.5	14.66	0.16	0.11
ALT	49.86	37	36.33	38	0.06	0.23
AST	47.2	40	42.0	48.5	0.54	0.79
Platelets	98.13	100.0	182.06	149.0	0.01	0.02

The other liver functions measured, including INR, total bilirubin, ALT, AST, GGT, and ALP, although, showed mild improvement among MRS group, it did not reach a significant level and were within the upper or lower reference normal physiological range.

The DSRS cohort only yielded a significant 2-year improvement in the platelet count, however, it was less than the improvement seen among MRS group, increasing from a mean value of 100 to 149.83 (*P* = 0.02). Despite a mild prolongation of PT and PTT values in this cohort, the liver function did not show noticeable changes (Table 1). Other liver function parameters did not show significant change and were within normal reference values before and after shunt surgery.

Following the multivariable analysis, a history of UVC was observed as a predictor of an obliterated Rex vein. Amongst our cohort, six patients had a history of UVC. Of this population, only two patients showed a patent Rex vein 33%, (*P* = 0.03). These patients were eligible for a MRS; however, one of them experienced shunt thrombosis immediately after surgery and proceeded to urgent DSRS, the other had reduced MRS flow at 6-month follow-up with further 2 episodes of variceal bleeding that resolved spontaneously.

Of the 23 patients who presented with EHPVO, 17 were without a history of UVC. 2 of these were lost to follow-up. Thirteen of the remining 15 had a patent Rex vein (86% presence rate compared with 33% in the group with a history of UVC) and received MRS—all were patent on follow-up.

## Discussion

This study investigated our 20-year, single-centre overall success rate of MRSs compared with the traditional DSRS used to treat EHPVO [24–26, 37]. Our findings confirm that children with EHPVO have a mild impairment in synthetic liver function and liver-dependent coagulation factors on presentation. This is manifested by low serum albumin, elevated INR, PT, PTT, and reduced fibrinogen. Following restoration of the hepatopetal blood flow to the liver with a MRS, a long-term improvement is seen in the levels of serum albumin, PTT, and INR. However, these findings are not observed in patients that received a DSRS even though both cohorts have shown no ongoing variceal bleeding post-shunt surgery. The negative impact of EHPVO on synthetic liver function was successfully reversed following MRS surgery. This has not been reproduced amongst DSRS group. Our findings highlight the risk of previous UVC on the patency of the Rex vein. This should facilitate the selection of patients when considered for either of these two procedures.

Twenty-one patients, with an average age of 5 years (range 2–12) were included in the study. This patient population is younger than most series in the literature that report ages at the time of shunt between 6 and 8 years of age [33, 38].

It is postulated that this is because our unit does not rely on lengthy variceal eradication programmes that require patients to attend on multiple occasions over many months and progress to shunt if this fail. This is because our patients often reside outside big centres and have difficulty accessing tertiary health care facilities should they need emergency management of a variceal bleed.

With respect to aetiology, 60% of our patients gave a history inclusive of one or more of the risk factors for EHPVO including UVC, abdominal tuberculosis with periportal lymphadenitis, omphalitis, and liver transplant. We could not identify any potential underlying aetiology in the remaining 40%. The aetiology of EHPVO influences the subsequent management of this condition; however, determining the predisposing factors for EHPVO remains a significant challenge. A study by Sarin and Agarwal [39] compared seven studies that assessed aetiology of portal vein thrombosis in infants and children. In most cases the cause could not be identified and where it could be, the majority of cases showed direct injury to the umbilical vascular system (omphalites and / or umbilical vein catheterization) or intrabdominal and umbilical sepsis to be a contributor. However, there also seemed to be a relationship between various causes suggesting a coexistent transient prothrombotic state that might result in extra hepatic portal vein thrombosis at the time of insult or shortly afterwards. With regard to umbilical vein catheterization, catheter dwell time over 3 days, catheter misplacement, trauma on catheter insertion site, type of solution infused and catheter related sepsis [39] all increase the risk for portal vein obstruction.

UVC and post liver transplant were the most prevalent causes in this cohort, with these presenting in six and five children, respectively. Moreover, we identified two children with neonatal omphalitis, two with periportal tuberculous lymphadenitis, in which one patient had a history of both TB and UVC. These findings are compatible with the current literature.

EHPVO can present as early as six weeks after birth as well as manifest in childhood. Clinical presentation depends on recent or chronic onset of clinical disease and age of presentation. The most common clinical features are haematemesis; often massive and usually not associated with major hepatocellular dysfunction. Gastrointestinal bleed is usually recurrent before a patient seeks medical attention. Patients can present with haematemesis and melena from conventional esophageal and / or gastric varices and can also bleed from ectopic varices or may present with obscure GI bleeding or bleeding from the biliary tract[40].

Hematemesis from bleeding varices was the commonest presenting symptom in our cohort (95%). Variceal bleeding is the most serious complication of portal hypertension in children [41, 42]. EHPVO may not be discovered until gastrointestinal haemorrhage develops [43].

Clinical splenomegaly was present in all our patients. Enlarged spleen is one of the classic findings of non-cirrhotic EHPH together with oesophageal varices and normal liver architecture [44]. Thus, the possibility of EHPVO should be suspected and a Doppler ultrasound performed in children and adolescents with upper gastrointestinal bleeding and/or isolated finding of splenomegaly during clinical examination.

Once the decision to perform shunt surgery is made, identifying the portal venous anatomy is the next critical step. This includes an accurate assessment of the portal venous flow proximal to the occlusion and the intra hepatic left portal outflow. The former is readily assessed by ultrasound and contrast-enhanced MRI. However, investigating the outflow of the intrahepatic left portal vein represents a diagnostic challenge. For this purpose, conventional angiography, Doppler ultrasound, contrast-enhanced CT, contrast-enhanced MRI and wedged hepatic vein portography (WHVP) has all been utilised with different accuracies and invasiveness.

Choosing the best modality depends on the availability and comfort of the radiologist interpreting the results. In our centre, WHVP is considered the gold standard for the assessment of Rex vein patency in preparation for MRS surgery. The sensitivity and specificity of WHVP of 80% and 95%, respectively, is comparable to results of a previous study by Lawson et al. (2011) [35].

The left internal jugular vein (IJV) lies lateral and anterior to both the internal and common carotid arteries and joins the subclavian vein to form the brachiocephalic vein [45]. The IJV is chosen as a vascular autograft over other conduit alternatives in the standard MRS technique [46]. For this reason, it is crucial that physicians avoid left sided central venous catheterization when resuscitating a child with EPHVO, and that anaesthetists are familiar with this as well.

In a large case series by Sherif et all, assessed children with EHPVO that were treated with a MRS using left internal jugular vein as interposition graft. A success rate of 91% was observed, over a median duration of 8 years (range 5.3–8.8 years) [47]. The study highlighted the superiority of venous autografts over synthetic grafts. This institution, thus, highly recommends using the left internal jugular vein as a shunt conduit in MRS surgery whenever this is technically possible.

We observed an overall success rate of shunt surgery 83% (19 patients of the 21 evaluated) across the two shunt groups, with MRS approach yielding a superior success rate compared to DSRS (93% versus 73%, respectively). This underpins our decision to preferentially adopt the MRS as the primary definitive technique, reserving the DSRS for patients where a MRS was not technically feasible or when the MRS fails.

It is of note that one child in our cohort was salvaged with a DSRS due to early MRS thrombosis. However, the reasons for this single operative failure of the MRS were thought to result from the surgical technique or an inadequate diameter Rex vein.

In this study, we confirmed the superiority of the MRS over DSRS in relieving variceal bleeding, with only one child out of fourteen (7%) who received MRS presenting with further variceal bleeding while two children out of seven (37%) who underwent DSRS presented with variceal bleeding at 2-year follow-up. Furthermore, we reported significant improvement in the platelet count in the children of both cohorts. However, compared with the increase post DSRS, Children with MRS showed a significant differential increase (*P* = 0.04). This is probably due to the significant reduction in the splenic size amongst MRS compared with DSRS [33], INR showed reduction from a mean of 1.9 pre MRS to a mean of 1.2 post-shunt. However, this did not reach a significant level (*P* = 0.24), and this might be due to the smaller size of the cohort, compared with almost unchanged value in children with DSRS.

We extended our study to include other parameters that reflects the synthetic liver function, in addition to the liver enzymes. PT and PTT showed a significant reduction in children with MRS. While PT went down in DSRS group, interestingly, PTT among DSRS children showed an increase. Although this was not statistically significant, it reflects procoagulant deficiencies which might be due to further shunting of the portal flow away from the liver.

Furthermore, a significant improvement in the serum albumin was noticed, 2 years post MRS. There was also a mild improvement in the serum fibrinogen, however, this was not statistically significant. These changes have not been seen amongst children underwent DSRS, which to the best of our knowledge has never been reported in literature by directly comparing MRS with DSRS.

Finally, liver transaminases have shown mild improvement 2 years after MRS. No changes appreciated among DSRS cohort. This improvement is believed to be due to the combined effects of resolution of the portal biliopathy, decompression of the portal circulation and restoration of hepatopetal flow.

Collectively, these findings demonstrate that both MRS and a portosystemic shunt are capable of effectively relieving symptoms in EHPVO. However, MRS demonstrated superiority regarding the alleviation of hypersplenism and offered additional metabolic benefits. These findings contrast with the recently published meta-analysis [38], which did not reveal superiority for either MRS or Portosystemic shunts, however, in this meta-analysis, only one study compared directly between both shunts and confirmed the better results of MRS regarding the metabolic outcome [33]. The paucity of well-conducted trials in this area justifies future multicentre studies and studies that examine long-term outcomes and directly compare MRS with DSRS.

Following the multivariable analysis on this patient cohort, a history of UVC was observed as a predictor of an obliterated Rex. Of the 6 patients with a history of UVC, 4 had an obliterated Rex vein, 1 had a failed MRS shunt most likely due to a compromised Rex vein, and 1 had initial reduced shut flow and subsequent bleeding which eventually settled. Thus, a previous neonatal umbilical catheter is a predictor of an obliterated Rex vein and possible MRS failure. However, due to the small numbers, it was impossible to comment on the role of UVC on the long-term patency of MRS. Similar studies have reported a success rate of MRS as low as 10% in patients with a history of UVC during the neonatal period [31, 48]. Guérin et al. recommended that MesoRex bypass be exclusively performed in patients without a previous history of UVC [31].

Collating this evidence, we hypothesise that the UVC is one of the primary causes of obliterated Rex vein in the paediatric population and hence, EHPVO. Moreover, it represents an independent risk factor for MRS failure. This arises following UVC-induced local injury and thrombosis in the Rex vein. The thrombus then propagates towards the left portal vein, before moving eventually to the main portal vein trunk. The clinical implications of these findings are vast for patients with a history of UVC and, given the poor performance of MRS in this instance, a more conservative approach might be warranted for disease management in this cohort. In other words, delaying MRS surgery until the patient’s endoscopic treatment fails may enable the underlying varices to be managed through an alternative measure as opposed to ending up with a DSRS.

## Conclusion

MRS has an improved long-term outcome in the management of extra hepatic portal vein obstruction, improves liver synthetic function and should be considered in children with favourable anatomy. DSRS does control variceal bleeding due to EHPVO but may have a negative effect on liver function in the long run and should only be considered when MRS is not technically feasible or as a salvage procedure when MRS fails.

## Limitations

Due to the retrospective nature of the study, there are a few limitations. A small sample size made it difficult to come up with a solid conclusion. Furthermore, Data concerning ALP and GGT was missing in several of the cohorts; thus, these parameters were excluded when comparing the shunts outcomes. This carries some limitations regarding subject to confounding and lack of standardisation. Moreover, the data collection was deficient in a few demographic criteria, including patient growth and nutritional status, limiting the statistical analysis.

## Data Availability

Data regading patients Growth and nutritional status were not consistently available for both cohorts, limiting the statistical analysis of these variables.
